# Supplementary material for: Structural basis for saxitoxin congener binding and neutralization by anuran saxiphilins
Source: Nat Commun. 2025 Apr 24;16:3885. doi: 10.1038/s41467-025-58903-2 (PMC12022044; doi:10.1038/s41467-025-58903-2)
Supplement: Supplementary file 1 — Supplementary Information [file 41467_2025_58903_MOESM1_ESM.pdf]

11 March 2025

Supplementary Material for

Structural basis for saxitoxin congener binding and neutralization by anuran saxiphilins

Sandra Zakrzewska[1], Samantha A. Nixon[1], Zhou Chen[1#], Holly S. Hajare[2], Elizabeth R. Park[2], John V. Mulcahy[2], Kandis M. Arlinghaus[3], Eduard Neu[4], Kirill Konovalov[4], Davide Provasi[4], Tod A. Leighfield[3], Marta Filizola[4], J. Du Bois[2], and Daniel L. Minor, Jr.[1, 5-9*]

[1]Cardiovascular Research Institute
[5]Department of Biochemistry and Biophysics
[6]Department of Cellular and Molecular Pharmacology
[7]California Institute for Quantitative Biomedical Research
[8]Kavli Institute for Fundamental Neuroscience
University of California, San Francisco, CA 94158-9001 USA

[9]Molecular Biophysics and Integrated Bio-imaging Division
Lawrence Berkeley National Laboratory, Berkeley, CA 94720 USA

[2]Department of Chemistry
Stanford University, Stanford, CA 94305 USA

[3]National Oceanic and Atmospheric Administration
National Centers for Coastal Ocean Science
Charleston, SC 29412 USA

[4]Department of Pharmacological Sciences
Ichan School of Medicine at Mount Sinai
New York, NY 10029 USA

*Correspondence to: daniel.minor@ucsf.edu

[#] Present address:
Department of Anatomy and Physiology
Shanghai Jiao Tong University School of Medicine
Shanghai, 200025, China

Table S1 Thermofluor data comparison.

Toxin	*Rc*Sxph ΔTm (°C)	ΔΔTm (°C)	n	*Np*Sxph ΔTm (°C)	ΔΔTm (°C)	n
**STX**	3.6 ± 0.2	-	8	3.2 ± 0.3	-	13
**dcSTX**	3.1 ± 0.1	-0.5 ± 0.2	4	2.2 ± 0.1	-1.0 ± 0.3	2
**GTX2/3**	2.7 ± 0.1	-0.9 ± 0.2	4	2.2 ± 0.1	-1.0 ± 0.3	4
**dcGTX2/3**	2.3 ± 0.1	-1.3 ± 0.2	5	1.4 ± 0.1	-1.8 ± 0.3	2
**GTX5**	3.7 ± 0.1	0.1 ± 0.2	4	3.3 ± 0.1	0.1 ± 0.3	2
**C1/C2**	2.6 ± 0.1	-1.0 ± 0.2	4	1.5 ± 0.2	-1.7 ± 0.4	4
**neoSTX**	0.7 ± 0.4	-2.9 ± 0.4	6	0.3 ± 0.1	-2.9 ± 0.3	4
**dc-neoSTX**	0.6 ± 0.3	-3.0 ± 0.4	5	0.4 ± 0.2	-2.8 ± 0.4	2
**GTX1/4**	-0.2 ± 0.1	-3.8 ± 0.2	4	-0.1 ± 0.1	-3.3 ± 0.3	3
**GTX6**	0.8 ± 0.3	-2.8 ± 0.4	6	0.2 ± 0.2	-3.0 ± 0.4	5

n, number of observations

$\Delta Tm = Tm_{Sxph+20\mu M\ toxin} - Tm_{Sxph}$

$\Delta\Delta Tm = Tm_{Sxph+20\mu M\ toxin} - Tm_{Sxph+20\mu M\ STX}$

ΔΔTm ranges are indicated as: White, STX; Green, ΔΔTm > -0.5 °C;
Yellow, -1.0 °C ≤ ΔΔTm ≤ -0.5 °C; Orange, -2.0 °C ≤ ΔΔTm < -1.0 °C; Red, ΔΔTm < -2.0 °C.

Errors are S.D.

Table S2 Competition Fluorescence Polarization (FPc) data comparison.

Toxin	*Rc*Sxph Kd (nM)	ΔΔG (kcal mol^{-1})	n	*Np*Sxph Kd (nM)	ΔΔG (kcal mol^{-1})	n
**STX**	8.9 ± 1.6	-	3	8.7 ± 1.7	-	4
**dcSTX**	20.3 ± 3.3	0.49	4	66 ± 16	1.19	4
**GTX2/3**	52.0 ± 8.3	1.04	3	133 ± 57	1.61	8
**dcGTX2/3**	180 ± 12	1.78	3	568 ± 68	2.47	3
**GTX5**	9.6 ± 0.8	0.04	3	7.3 ± 1.8	-0.10	4
**C1/C2**	97.0 ± 8.3	1.41	3	186 ± 46	1.81	6
**neoSTX**	>5000	>3.7	3	>5000	>3.7	5
**dc-neoSTX**	>5000	>3.7	3	>5000	>3.7	6
**GTX1/4**	>5000	>3.7	3	>5000	>3.7	3
**GTX6**	1516 ± 222	3.04	3	1239 ± 578	2.94	4

n, number of observations

Kd, dissociation constant

$\Delta\Delta G = RT \ln(Kd_{toxin}/Kd_{STX})$ T = 298 K

ΔΔG ranges are indicated as: White, STX; Green, ΔΔG < 1.0 kcal mol^{-1}; Yellow, 1.0 ≤ ΔΔG < 2.0 kcal mol^{-1}; Orange, 2.0 ≤ ΔΔG ≤ 3.0 kcal mol^{-1}; Red, ΔΔG > 3.0 kcal mol^{-1}

Errors are S.D.

Table S3 Radioligand receptor binding (RBA) data comparison.

Toxin	*Rc*Sxph Kd (nM)	ΔΔG (kcal mol^{-1})	n	*Np*Sxph Kd (nM)	ΔΔG (kcal mol^{-1})	n	Rat brain homogenate Kd (nM)	ΔΔG (kcal mol^{-1})	n
**STX**	7.1 ± 1.1	-	5	6.1 ± 1.3	-	7	1.5 ± 0.2	-	10
**dcSTX**	20 ± 4.0	0.61	5	40 ± 10	1.11	7	2.8 ± 0.3	0.37	2
**GTX2/3**	48 ± 10	1.13	5	52 ± 13	1.27	7	4.8 ± 1.1	0.69	4
**dcGTX2/3**	159 ± 6.0	1.84	5	396 ± 168	2.47	6	20 ± 4.0	1.53	4
**GTX5**	5.0 ± 1.6	-0.21	6	3.2 ± 2.3	-0.38	5	326 ± 72	3.19	3
**C1/C2**	106 ± 7.0	1.60	3	255 ± 16	2.21	3	221 ± 91	2.96	3
**neoSTX**	>1000	>3.0	2	>1000	>3.0	3	0.5 ± 0.1	-0.65	2
**dc-neoSTX**	>1000	>3.0	2	>1000	>3.0	4	61 ± 39	2.19	4
**GTX1/4**	>1000	>3.0	2	>1000	>3.0	3	3.2 ± 1.0	0.45	4
**GTX6**	>1000	>3.0	5	>1000	>3.0	7	88 ± 5.1	2.41	3

n, number of observations

$\Delta\Delta G = RT \ln(Kd_{toxin}/Kd_{STX})$ T = 298 K

ΔΔG ranges are indicated as: White, STX; Green, $\Delta\Delta G < 1.0$ kcal mol^{-1}; Yellow, $1.0 \leq \Delta\Delta G < 2.0$ kcal mol^{-1}; Orange, $2.0 \leq \Delta\Delta G \leq 3.0$ kcal mol^{-1}; Red, $\Delta\Delta G > 3.0$ kcal mol^{-1}

Errors are S.D.

Table S4 Crystallographic data collection and refinement statistics

	*Np*Sxph:dcSTX (co-crystal) PDB: 8V68	*Np*Sxph:GTX2 (co-crystal) PDB: 8V69	*Np*Sxph:dcGTX2 (co-crystal) PDB: 8V65	*Np*Sxph:GTX5 (co-crystal) PDB: 8V66	*Np*Sxph:C1 (co-crystal) PDB: 8V67
**Data Collection**					
Space group	R3	R3	R3	R3	R3
Cell dimensions a/b/c (Å)	229.47, 229.47, 67.61	229.74, 229.74, 67.87	229.67, 229.67, 67.71	229.16, 229.16, 67.50	229.78, 229.78, 67.39
α/β/γ (°)	90, 90, 120	90, 90, 120	90, 90, 120	90, 90, 120	90, 90, 120
Resolution (Å)	43.37-1.9 (1.968-1.9)	43.42-1.95 (2.02-1.95)	43.4-1.8 (1.864-1.8)	43.31-1.9 (1.968-1.9)	43.43-1.90 (1.968-1.90)
Rmerge (%)	0.1038 (3.545)	0.09641 (2.338)	0.1286 (5.018)	0.1362 (2.498)	0.2557 (3.836)
I / σ I	12.65 (1.03)	9.91 (0.81)	9.74 (0.66)	8.15 (1.19)	6.51 (0.69)
CC(1/2)	0.999 (0.49)	0.999 (0.41)	0.998 (0.394)	0.998 (0.58)	0.997 (0.356)
Completeness (%)	99.57 (95.89)	99.98 (100.00)	99.97 (99.90)	99.98 (99.99)	99.92 (99.50)
Redundancy	15.1 (15.5)	10.0 (10.2)	14.9 (14.7)	15.1 (15.6)	30.2 (31.0)
Total reflections	1583855 (161838)	972728 (98797)	1843446 (181645)	1577994 (162070)	3161523 (322475)
Unique reflections	104618 (10454)	97333 (9717)	123415 (12335)	104167 (10420)	104543 (10419)
Wilson B-factor	43.54	49.43	43.13	42.78	44.84
Wavelength (Å)	1.116	1.116	1.116	1.116	1.116
**Refinement**					
R_{work} / R_{free} (%)	19.65/22.43	18.68/21.16	19.88/21.82	19.00/21.82	20.70/22.96
No. of chains in AU	1	1	1	1	1
No. of protein atoms	6392	6381	6373	6402	6382
No. of ligand atoms	34	42	16	41	46
No. of water atoms	400	273	428	378	291
RMSD bond lengths (Å)	0.003	0.014	0.004	0.007	0.009
RMSD angles (°)	0.57	1.24	0.68	0.90	1.03
Ramachandran favored/allowed/outliers (%)	96.95/2.93/0.12	95.96/3.92/0.12	96.93/2.82/0.25	96.21/3.54/0.24	95.95/3.93/0.12

Table S5 *Hs*Na$_V$1.4 and *Pt*Na$_V$1.4 toxin responses.

Toxin	*Hs*Na$_V$1.4			*Pt*Na$_V$1.4		
	IC$_{50}$ (nM)	IC$_{90}$ (nM)	n	IC$_{50}$ (nM)	IC$_{90}$ (nM)	n
**STX**	3.0 ± 1.6	50	9	12.6 ± 1.4*	100	6
**dcSTX**	14.7 ± 8.7	200	4	144.4 ± 29.7	800	6
**GTX2/3**	6.8 ± 1.1	60	8	27.4 ± 2.1	200	6
**dcGTX2/3**	31.8 ± 10.6	300	6	N/A	N/A	N/A
**GTX5**	335.3 ± 55.4	2700	8	2290 ± 776	>10,000	6
**C1/C2**	151.5 ± 37.0	1000	5	N/A	N/A	N/A

IC$_{50}$, half-maximal inhibitory concentration

IC$_{90}$, concentration required to block 90% of the current

n, number of cells

Errors are S.D.

*data taken from [1]

Figure S1 **Zakrzewska *et al.***

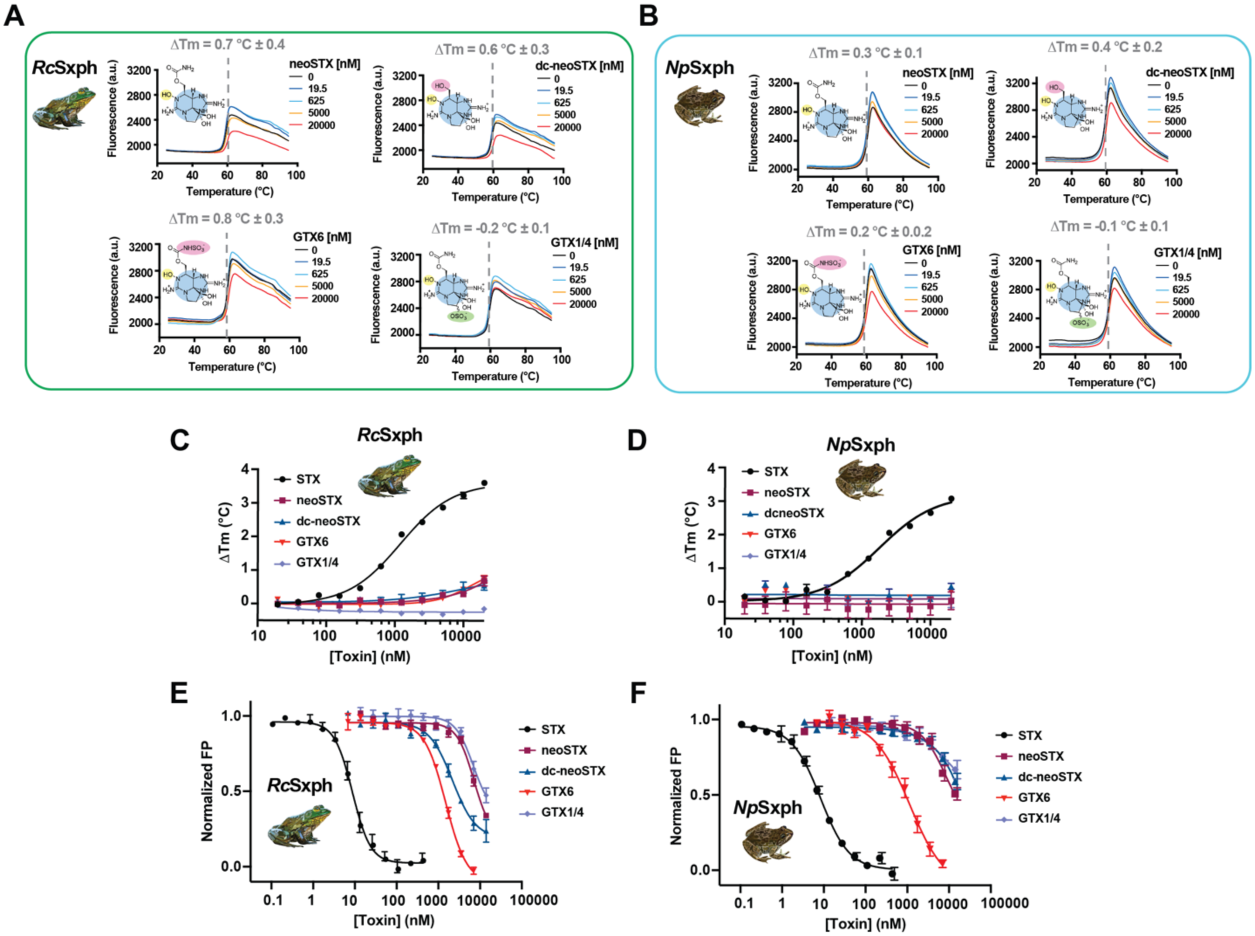

Figure S1 *Rc*Sxph and *Np*Sxph poorly interact with neo-STX congeners. A, and **B,** Exemplar TF assay results for **A,** *Rc*Sxph and **B,** *Np*Sxph in the presence of the indicated concentrations of neoSTX, dc-neoSTX, GTX6, and GTX1/4. Toxin concentrations are 0 nM (black), 19.5 nM (blue), 625 nM (cyan), 5,000 nM (orange), and 20,000 nM (red). **C,** and **D,** Temperature dependence of ΔTm as a function of toxin concentration for TF assays of **C,** *Rc*Sxph and **D,** *Np*Sxph with STX (black circles), neoSTX (maroon squares), dc-neoSTX (blue triangles), GTX6 (red inverted triangles), and GTX1/4 (lavender diamonds). **E,** and **F,** FP competition binding assays for **E,** *Rc*Sxph and **F,** *Np*Sxph with STX (black circles), neoSTX (maroon squares), dc-neoSTX (blue triangles), GTX6 (red inverted triangles), and GTX1/4 (lavender diamonds). Error bars are S.E.M. n= 2-8. Source data are provided as a Source Data file. Specific 'n' values are contained therein.

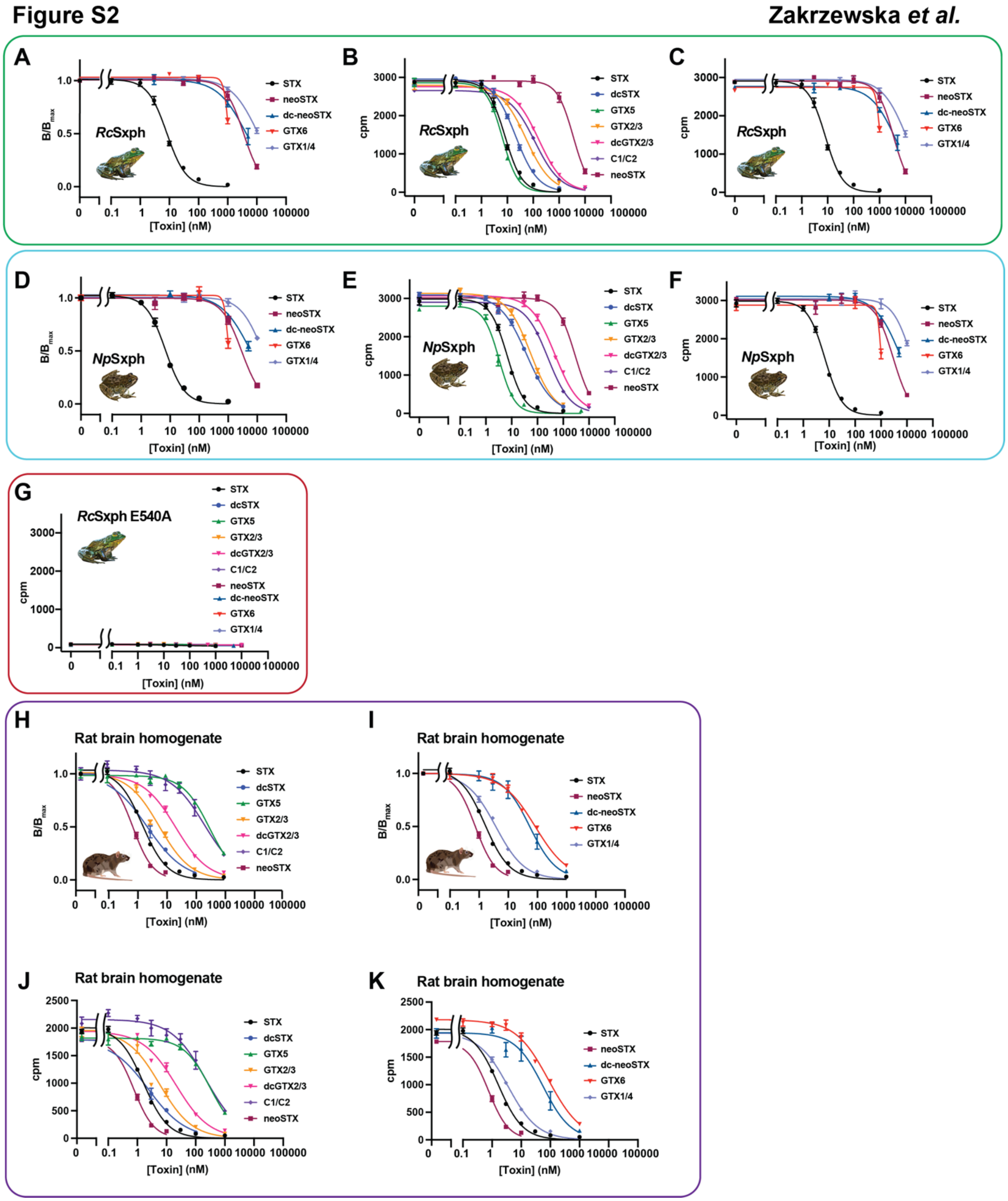

Figure S2 Receptor binding assay comparisons. A, *Rc*Sxph B/B_{max} normalized RBA data for the indicated toxin, where B represents the bound [^{3}H]STX in the sample and B_{max} is the maximum binding of [^{3}H]STX in the absence of competing unlabeled toxin. **B,** and **C**, Raw radioactive counts per minute (cpm) for *Rc*Sxph RBAs. **D,** *Np*Sxph normalized RBA data for the indicated toxins. **E,**

and **F**, Raw cpm for *Np*Sxph RBAs. **G,** Raw cpm for *Rc*Sxph E540A RBA. **H,** and **I,** Normalized RBA data for rat brain homogenate binding of **H,** STX, STX congeners, and neo-STX and **I,** STX, neo-STX, and neo-STX congeners. **J,** and **K**, Raw cpm for rat brain homogenate RBAs. In all panels toxins indicated as STX (black circles), dcSTX (blue circles), GTX5 (green triangles), GTX2/3 (orange inverted triangles), dcGTX2/3 (magenta inverted triangles), C1/C2 (lavender diamonds), neoSTX (dark red squares), dc-neoSTX (blue triangles), GTX6 (inverted red triangles), and GTX1/4 (light purple diamonds). Error bars are S.E.M. n=2-10 Source data are provided as a Source Data file. Specific 'n' values are contained therein.

Figure S3 **Zakrzewska *et al.***

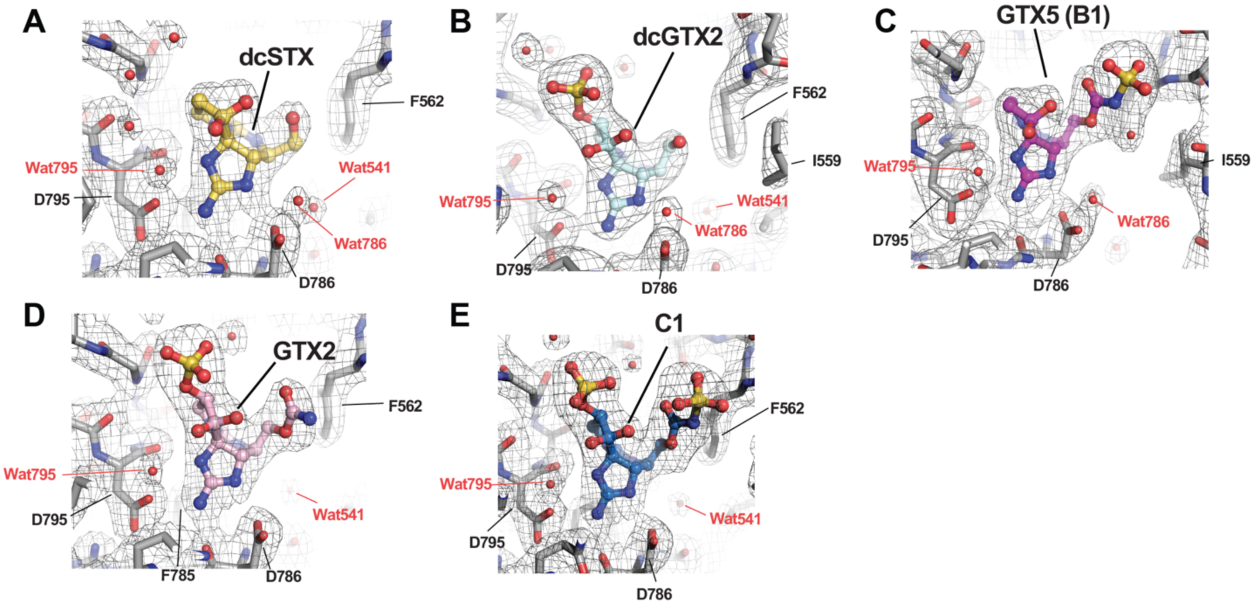

Figure S3 Structures of *Np*Sxph:STX congener complexes. A-E, Exemplar electron density (1σ) for **A,** *Np*Sxph:dcSTX (yellow). **B,** *Np*Sxph:dcGTX2 (cyan). **C,** *Np*Sxph:GTX5 (B1) (magenta). **D,** *Np*Sxph:GTX2 (light pink). **E,** *Np*Sxph:C1 (marine). *Np*Sxph is light grey. Water molecules (red) are shown as spheres. Select residues are labeled.

Figure S4 **Zakrzewska *et al.***

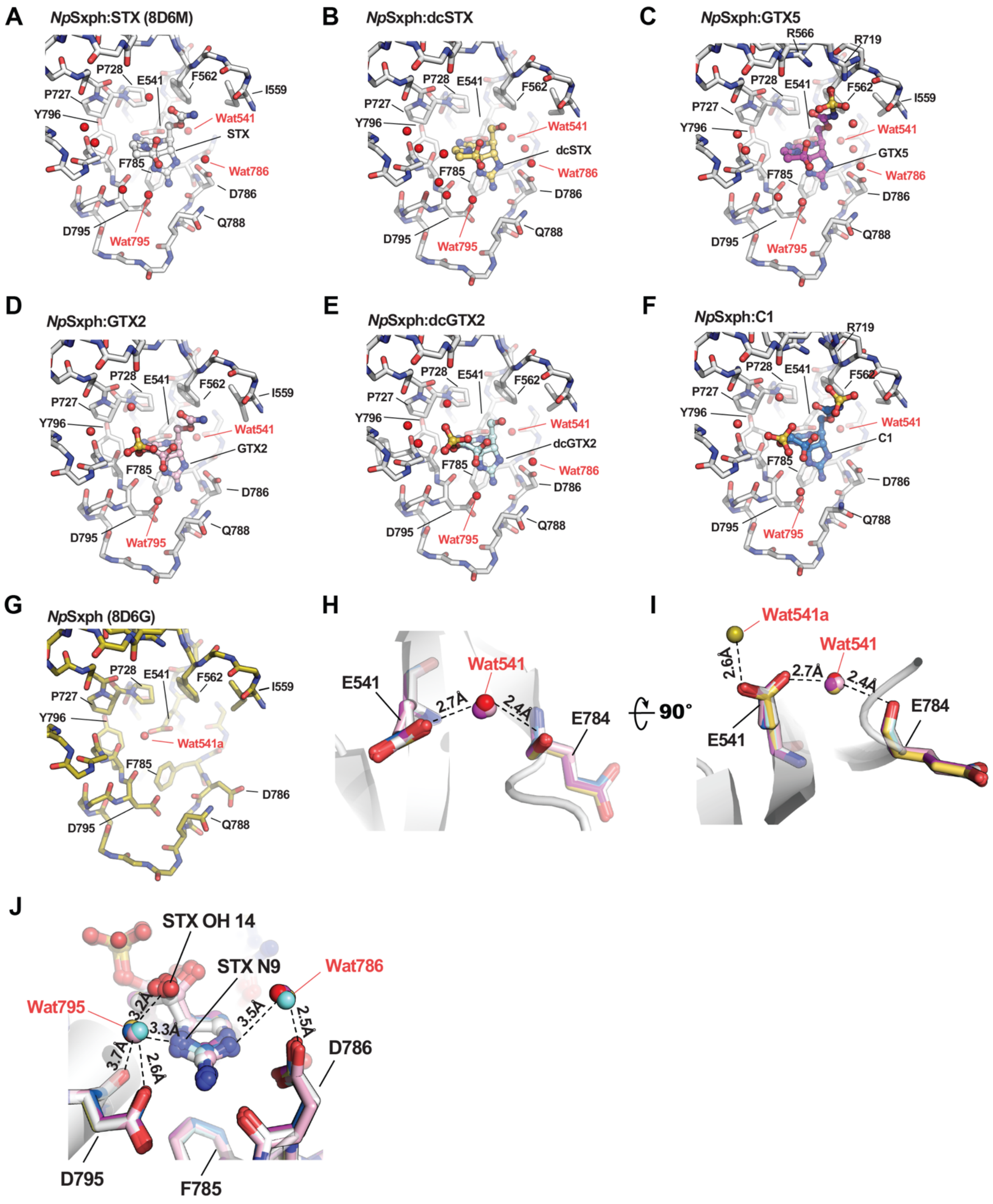

Figure S4 *Np*Sxph STX binding pocket water network structure. A-F, *Np*Sxph:toxin complexes (white) showing the positions of crystallographic water molecules (red spheres) in the

toxin binding pocket for **A,** *Np*Sxph:STX (white) (PDB:8D6M) [2]. **B,** *Np*Sxph:dcSTX (yellow). **C,** *Np*Sxph:GTX5 (magenta). **D,** *Np*Sxph:GTX2 (light pink). **E,** *Np*Sxph:dcGTX2 (cyan). **F,** *Np*Sxph:C1 (marine). **G,** apo-*Np*Sxph (PDB:8D6G) (yellow orange) [2]. **H,** Close up view of Wat541 and coordinating residues Glu541 and Glu784 for *Np*Sxph:STX (white), *Np*Sxph:dcSTX (yellow), *Np*Sxph:dcGTX2 (cyan), *Np*Sxph:GTX5 (magenta), *Np*Sxph:GTX2 (light pink) and, *Np*Sxph:C1 (marine). **I,** Close up view of the positions of Wat541a and Wat541. Colors are as in 'H'. apo-*Np*Sxph (PDB:8D6G) (yellow orange). **J,** Close up view of the positions of Wat786 and Wat795 for *Np*Sxph:STX (white), *Np*Sxph:dcSTX (yellow), *Np*Sxph:dcGTX2 (cyan), *Np*Sxph:GTX5 (magenta), *Np*Sxph:GTX2 (light pink) and, *Np*Sxph:C1 (marine). STX positions of N9 and OH 14 are indicated.

Figure S5

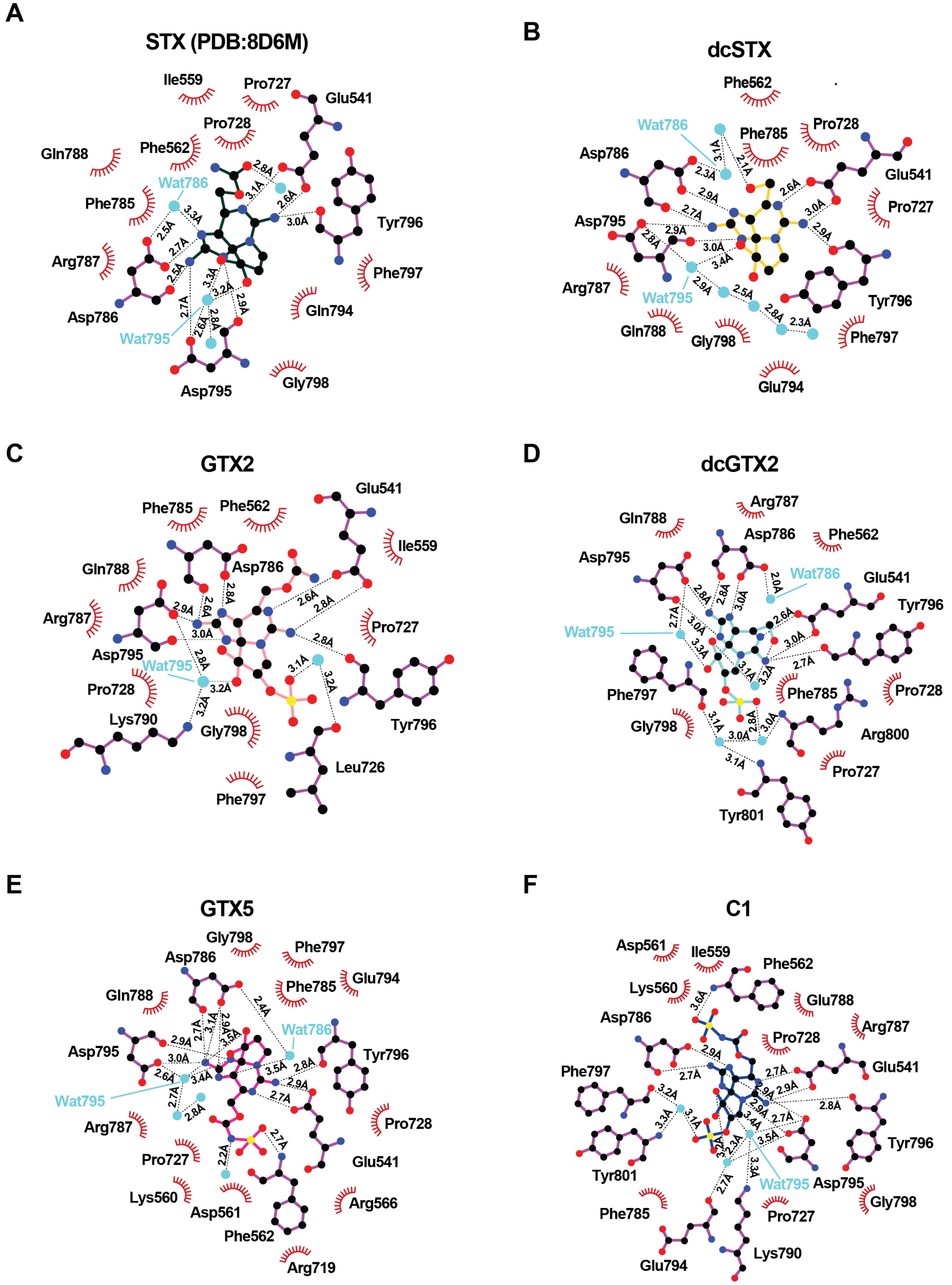
A
STX (PDB:8D6M)
Ile559
Pro727
Glu541
Pro728
Gln788
Phe562
Wat786
Phe785
Tyr796
Arg787
Phe797
Asp786
Gln794
Wat795
Asp795
Gly798
B
dcSTX
Phe562
Wat786
Phe785
Pro728
Asp786
Glu541
Pro727
Asp795
Arg787
Wat795
Tyr796
Gln788
Gly798
Phe797
Glu794
C
GTX2
Glu541
Phe785
Phe562
Ile559
Gln788
Asp786
Arg787
Pro727
Asp795
Wat795
Pro728
Lys790
Gly798
Tyr796
Leu726
Phe797
D
dcGTX2
Arg787
Gln788
Asp786
Phe562
Asp795
Wat786
Glu541
Tyr796
Wat795
Phe797
Phe785
Pro728
Gly798
Arg800
Pro727
Tyr801
E
GTX5
Gly798
Phe797
Asp786
Phe785
Glu794
Gln788
Wat786
Asp795
Tyr796
Wat795
Arg787
Pro728
Pro727
Glu541
Lys560
Asp561
Arg566
Phe562
Arg719
F
C1
Asp561
Ile559
Phe562
Lys560
Glu788
Asp786
Arg787
Pro728
Glu541
Phe797
Tyr796
Tyr801
Wat795
Asp795
Gly798
Phe785
Pro727
Glu794
Lys790

Figure S5 *Np*Sxph:STX congener interactions. A-F, LIGPLOT [3] diagrams showing interactions (5.0Å cutoff) for: **A**, *Np*Sxph:STX (PDB:8D6M) [2]. **B,** *Np*Sxph:dcSTX (yellow). **C,** *Np*Sxph:GTX2 (light pink). **D,** *Np*Sxph:dcGTX2 (cyan). **E,** *Np*Sxph:GTX5 (magenta). **F,** *Np*Sxph:C1 (marine).

Figure S6 **Zakrzewska *et al.***

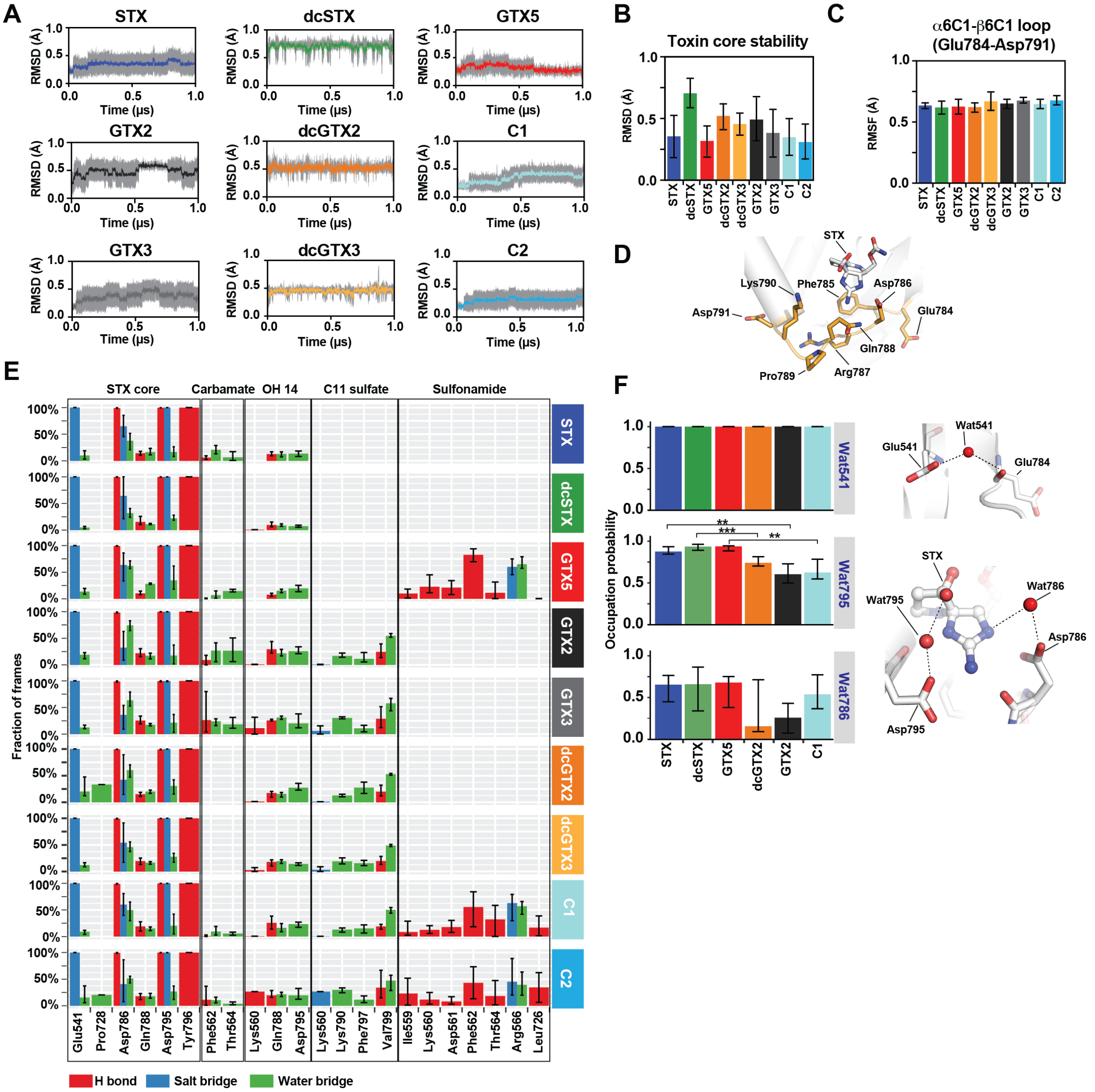

Figure S6 *Np*Sxph:toxin simulations highlight ligand stability and effects on water network. **A,** Time series of RMSD calculated from the crystallographic pose for non-hydrogen ligand atoms common to all STX congeners during 1 μs simulations (n=5). **B,** Mean and standard deviation of the average RMSD for the STX core atoms over the entire trajectory. **C,** Mean and standard deviation of the average RMSF for *Np*Sxph α6C1-β6C1 loop residues Cα atoms during 1 μs simulations (n=5). **D,** *Np*Sxph α6C1-β6C1 loop (orange) position in the *Np*Sxph:STX complex (PDB 8D6M)[2]. **E,** Structural interaction fingerprint (SIFt) analyses of *Np*Sxph complexes grouped by interaction type, protein residue, and interacting toxin moiety. **F,** (left) Mean probability of occupancy of the indicated hydration sites (Wat541, Wat795, and Wat786) for all simulated

systems. For Wat795, a Welch two-sample two-sided t-test reveals significant differences in mean occupancy between dcGTX2 and dcSTX, GTX2 and STX, and GTX5 and C1, with p-values of 0.00219, 0.0137, and 0.0129, respectively. For Wat786, only the comparison between GTX2 and STX shows a significant, albeit weak, difference, (p-value = 0.0234). Comparisons between dcGTX2 and dcSTX and between GTX5 and C1 yield p-values > 0.1. (right) Locations of Wat541, Wat795, and Wat786 in the *Np*Sxph:STX structure (PDB: 8D6M)[2]. Dashed lines indicate hydrogen bonds. Error bars represent S.D. of the mean fraction of frames over five trajectories. ** $p < 0.02$, *** $p < 0.005$. Source data are provided as a Source Data file.

Figure S7 **Zakrzewska *et al.***

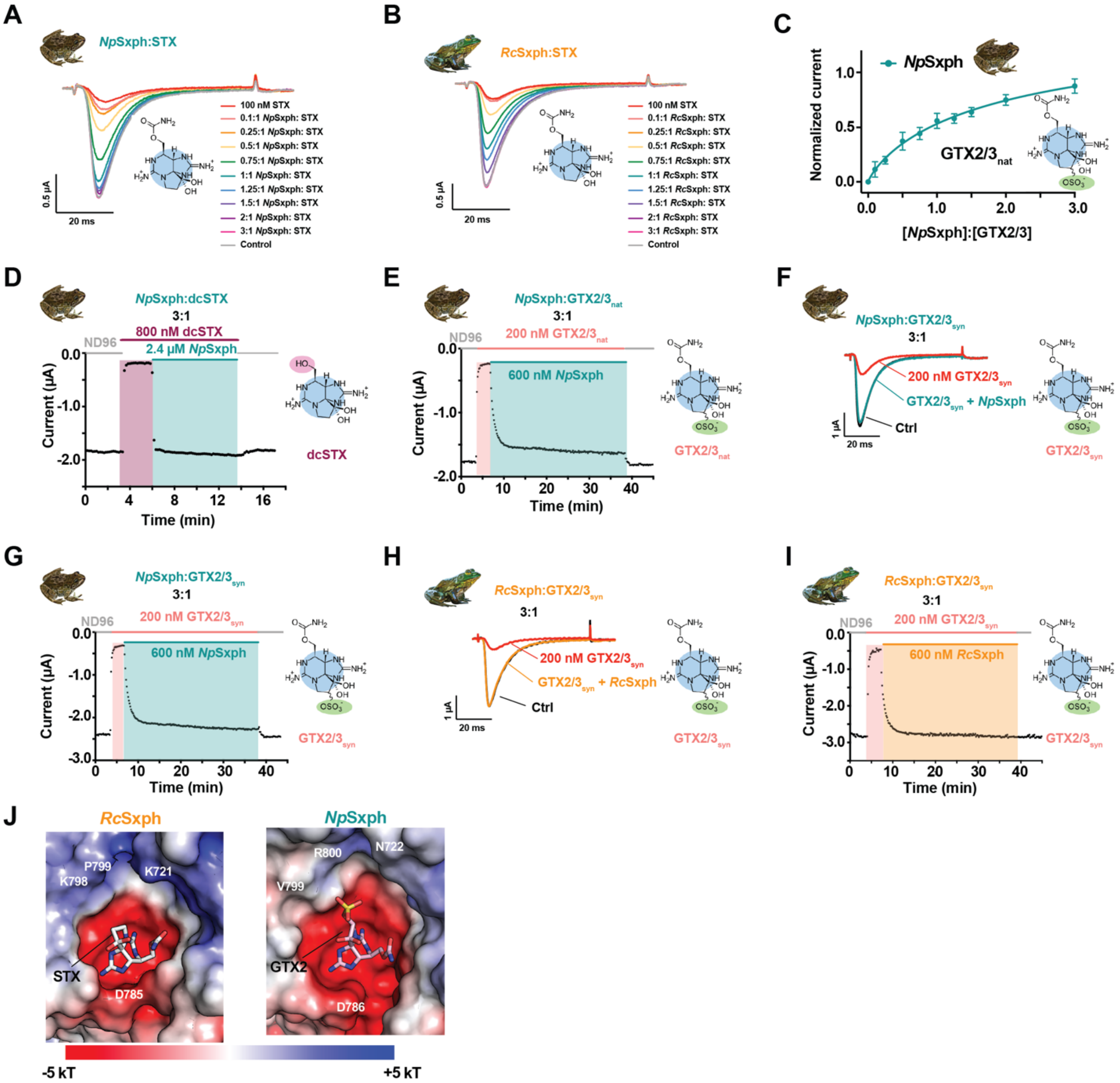

Figure S7 Sxphs rescue *Pt*Na_V1.4 from toxin block. A and **B,** Exemplar two electrode voltage clamp (TEVC) recordings of *Pt*Na_V1.4 expressed in *Xenopus* oocytes in the presence of 100 nM STX, the indicated Sxph:STX ratios, or control conditions for **A,** *Np*Sxph and **B,** *Rc*Sxph. **C,** [*Np*Sxph]:[GTX2/3] dose-response curve in the presence of 200 nM GTX2/3. **D,** and **E,** *Pt*Na_V1.4 responses to application of **D,** 800 nM dcSTX (purple) and **E,** 200 nM $GTX2/3_{nat}$ (light red) and 3:1 *Np*Sxph:toxin application (bluegreen). **F,** Exemplar TEVC recordings of *Pt*Na_V1.4 expressed in *Xenopus* oocytes in the presence of 200 nM $GTX2/3_{syn}$ and 3:1 *Np*Sxph:$GTX2/3_{syn}$. **G,** *Pt*Na_V1.4 response to application of 200 nM $GTX2/3_{syn}$ (light red) and 3:1 *Np*Sxph:toxin application (bluegreen). **H,** Exemplar TEVC recordings of *Pt*Na_V1.4 expressed in *Xenopus*

oocytes in the presence of 200 nM GTX2/3_{syn} and 3:1 *Rc*Sxph:GTX2/3_{syn}. **I,** *Pt*Na_V1.4 response to application of 200 nM GTX2/3_{syn} (light red) and 3:1 *Rc*Sxph:toxin application (orange). **J,** Comparisons of the electrostatic surface potentials of the toxin binding pocket from *Rc*Sxph:STX (PDB:6O0F)[4] with *Np*Sxph:GTX2 (PDB:8V69)[2]. STX and GTX2 are shown as sticks. Source data are provided as a Source Data file.

Figure S8 **29 aug 24** **Zakrzewska *et al.***

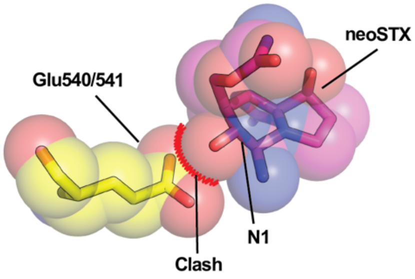

Figure S8 Model of Sxph STX binding pocket:neoSTX clash. Superposition of neoSTX on STX in the *Rc*Sxph:STX complex (PDB:6O0F)[4] indicates clash of the neoSTX N1 hydroxyl with the conserved glutamate (*Rc*Sxph540, *Np*Sxph541).

References

1. Abderemane-Ali, F. et al. Evidence that toxin resistance in poison birds and frogs is not rooted in sodium channel mutations and may rely on "toxin sponge" proteins. *J Gen Physiol* **153**(2021).

2. Chen, Z. et al. Definition of a saxitoxin (STX) binding code enables discovery and characterization of the anuran saxiphilin family. *Proc Natl Acad Sci U S A* **119**, e2210114119 (2022).

3. Wallace, A.C., Laskowski, R.A. & Thornton, J.M. LIGPLOT: a program to generate schematic diagrams of protein-ligand interactions. *Protein Eng* **8**, 127-34 (1995).

4. Yen, T.-J., Lolicato, M., Thomas-Tran, R., Du Bois, J. & Minor, D.L., Jr. Structure of the Saxiphilin:saxitoxin (STX) complex reveals a convergent molecular recognition strategy for paralytic toxins. *Sci Adv* **5**(2019).
