# Supplementary material for: Structural basis for saxitoxin congener binding and neutralization by anuran saxiphilins
Source: Nat Commun. 2025 Apr 24;16:3885. doi: 10.1038/s41467-025-58903-2 (PMC12022044; doi:10.1038/s41467-025-58903-2)
Supplement: Supplementary file 2 — Description of Additional Supplementary Files [file 41467_2025_58903_MOESM2_ESM.pdf]

Description of Additional Supplementary Files

File Name: Supplementary Movie 1
Description: **Conformational changes between *Np*Sxph:STX congener structures.** Morph between the apo-*Np*Sxph (PDB:8D6G)[2] and each *Np*Sxph:STX congener structures showing the toxin binding site.

NOTE: Reference is to the reference list in supplementary.
