# Supplementary material for: Structural basis for saxitoxin congener binding and neutralization by anuran saxiphilins
Source: Nat Commun. 2025 Apr 24;16:3885. doi: 10.1038/s41467-025-58903-2 (PMC12022044; doi:10.1038/s41467-025-58903-2)
Supplement: Supplementary file 4 — Reporting Summary [file 41467_2025_58903_MOESM4_ESM.pdf]

nature portfolio

Corresponding author(s): Daniel L. Minor, Jr., Ph.D.

Last updated by author(s): Mar 10, 2025

Reporting Summary

Nature Portfolio wishes to improve the reproducibility of the work that we publish. This form provides structure for consistency and transparency in reporting. For further information on Nature Portfolio policies, see our Editorial Policies and the Editorial Policy Checklist.

Statistics

For all statistical analyses, confirm that the following items are present in the figure legend, table legend, main text, or Methods section.

n/a	Confirmed	
☐	☒	The exact sample size (*n*) for each experimental group/condition, given as a discrete number and unit of measurement
☐	☒	A statement on whether measurements were taken from distinct samples or whether the same sample was measured repeatedly
☒	☐	The statistical test(s) used AND whether they are one- or two-sided<br>*Only common tests should be described solely by name; describe more complex techniques in the Methods section.*
☐	☒	A description of all covariates tested
☒	☐	A description of any assumptions or corrections, such as tests of normality and adjustment for multiple comparisons
☐	☒	A full description of the statistical parameters including central tendency (e.g. means) or other basic estimates (e.g. regression coefficient) AND variation (e.g. standard deviation) or associated estimates of uncertainty (e.g. confidence intervals)
☒	☐	For null hypothesis testing, the test statistic (e.g. *F*, *t*, *r*) with confidence intervals, effect sizes, degrees of freedom and *P* value noted<br>*Give P values as exact values whenever suitable.*
☒	☐	For Bayesian analysis, information on the choice of priors and Markov chain Monte Carlo settings
☒	☐	For hierarchical and complex designs, identification of the appropriate level for tests and full reporting of outcomes
☒	☐	Estimates of effect sizes (e.g. Cohen's *d*, Pearson's *r*), indicating how they were calculated

Our web collection on statistics for biologists contains articles on many of the points above.

Software and code

Policy information about availability of computer code

Data collection	XDS Kabsch, W. Xds. Acta Crystallogr D Biol Crystallogr 66, 125-32 (2010)<br>Aimless Evans, P.R. & Murshudov, G.N. How good are my data and what is the resolution? Acta Crystallogr D Biol Crystallogr 69, 1204-14 (2013)<br>Phenix Adams, P.D. et al. PHENIX: a comprehensive Python-based system for macromolecular structure solution. Acta Crystallogr D Biol Crystallogr 66, 213-21 (2010).<br><br>Two electrode voltage clamp data were acquired using pClamp software 10.9 (Molecular Devices).<br>Whole-cell patch clamp data were acquired using Sophion software (Sophion Bioscience).
Data analysis	TF, FPc and RBA data were analyzed using GraphPad Prism 10.1 (GraphPad Software).<br>ITC data analysis was performed using MicroCal PEAQ-ITC Analysis Software (Malvern Panalytical).<br>COOT Emsley, P. & Cowtan, K. Coot: model-building tools for molecular graphics. Acta Crystallogr D Biol Crystallogr 60, 2126-32 (2004).<br>MolProbity Williams, C.J. et al. MolProbity: More and better reference data for improved all-atom structure validation. Protein Sci 27, 293-315 (2018)<br>CHARMM-GUI Jo, S., Kim, T., Iyer, V.G. & Im, W. CHARMM-GUI: a web-based graphical user interface for CHARMM. J Comput Chem 29, 1859-65 (2008)<br>Prime (Schrödinger Suite 2024) Jacobson, M.P. et al. A hierarchical approach to all-atom protein loop prediction. Proteins 55, 351-67 (2004)<br>AMBER TianC. et al. ff19SB: Amino-Acid-Specific Protein Backbone Parameters Trained against Quantum Mechanics Energy Surfaces in Solution. J Chem Theory Comput 16, 528-552 (2020).<br>Sage (OpenFF 2.1.0) Boothroyd, S. et al. Development and Benchmarking of Open Force Field 2.0.0: The Sage Small Molecule Force Field. J

Chem Theory Comput 19, 3251-3275 (2023).
GROMACS 2024.2 Abraham, M.J. et al. GROMACS: High performance molecular simulations through multi-level parallelism from laptops to supercomputers. SoftwareX 1-2, 19-25 (2015)
EWALD Darden, T., York, D. & Pedersen, L. Particle Mesh Ewald - an N.Log(N) Method for Ewald Sums in Large Systems. Journal of Chemical Physics 98, 10089-10092 (1993)

Two electrode voltage clamp data were analyzed with Clampfit 11.0 (Molecular Devices).
Whole-cell patch clamp data were analyzed with Sophion Analyzer software (Sophion Bioscience).

MDTraj McGibbon, R.T. et al. MDTraj: A Modern Open Library for the Analysis of Molecular Dynamics Trajectories. Biophys J 109, 1528-32 (2015).
PLIP v2.3.0 Salentin, S., Schreiber, S., Haupt, V.J., Adasme, M.F. & Schroeder, M. PLIP: fully automated protein-ligand interaction profiler. Nucleic Acids Res 43, W443-7 (2015).

For manuscripts utilizing custom algorithms or software that are central to the research but not yet described in published literature, software must be made available to editors and reviewers. We strongly encourage code deposition in a community repository (e.g. GitHub). See the Nature Portfolio guidelines for submitting code & software for further information.

Data

Policy information about availability of data

All manuscripts must include a data availability statement. This statement should provide the following information, where applicable:
- Accession codes, unique identifiers, or web links for publicly available datasets
- A description of any restrictions on data availability
- For clinical datasets or third party data, please ensure that the statement adheres to our policy

Coordinates and structure factors and for NpSxph:dcSTX (PDB:8V68), NpSxph:GTX2 (PDB:8V69), NpSxph:dcGTX2 (PDB:8V65), NpSxph:GTX5 (PDB:8V66), and NpSxph:C1 (PDB:8V67) are deposited with the RCSB and will be released upon publication.
The source data underlying Figures 1B-E, 2A-I, 3F, 5A-G, 6A-B, 6C-E, and Supplementary Figures S1A-F, S2A-K, S6A-C, S6E-F, and S7A-I are provided as a Source Data file.
Source data for previously published PDB codes are: 8D6G [https://doi.org/10.2210/pdb8D6G/pdb], 8D6M [https://doi.org/10.2210/pdb8D6M/pdb], and 6J8G [https://doi.org/10.2210/pdb6J8G/pdb]
Requests for material should be sent to D.L.M.

Research involving human participants, their data, or biological material

Policy information about studies with human participants or human data. See also policy information about sex, gender (identity/presentation), and sexual orientation and race, ethnicity and racism.

Reporting on sex and gender	N/A
Reporting on race, ethnicity, or other socially relevant groupings	N/A
Population characteristics	N/A
Recruitment	N/A
Ethics oversight	N/A

Note that full information on the approval of the study protocol must also be provided in the manuscript.

Field-specific reporting

Please select the one below that is the best fit for your research. If you are not sure, read the appropriate sections before making your selection.

[x] Life sciences [ ] Behavioural & social sciences [ ] Ecological, evolutionary & environmental sciences

For a reference copy of the document with all sections, see nature.com/documents/nr-reporting-summary-flat.pdf

Life sciences study design

All studies must disclose on these points even when the disclosure is negative.

Sample size	Sample sizes were determined according to field standards.
Data exclusions	No data were excluded from the analyses.
Replication	All experiments were performed with independent replicates. For two electrode voltage-clamp studies, the results are from at least two

	independent oocyte batches. n = 7-8 oocytes. For the whole-cell patch clamp, the results are from at least four independent CHO cells. n=4-7. ('n' value is shown for each panel). All replication attempts were successful.
Randomization	Randomization is not relevant to binding and crystallographic studies.<br>Electrophysiological studies are inherently randomized as there is no bias in selecting cells for recording.
Blinding	Blinding is not relevant to this study as no subjective allocation was involved.

Reporting for specific materials, systems and methods

We require information from authors about some types of materials, experimental systems and methods used in many studies. Here, indicate whether each material, system or method listed is relevant to your study. If you are not sure if a list item applies to your research, read the appropriate section before selecting a response.

Materials & experimental systems

n/a	Involved in the study	
x		Antibodies
	x	Eukaryotic cell lines
x		Palaeontology and archaeology
	x	Animals and other organisms
x		Clinical data
x		Dual use research of concern
x		Plants

Methods

n/a	Involved in the study	
x		ChIP-seq
x		Flow cytometry
x		MRI-based neuroimaging

Eukaryotic cell lines

Policy information about cell lines and Sex and Gender in Research

Cell line source(s)	Chinese hamster ovary (CHO) cells stably expressing the alpha-subunit of human NaV1.4 sodium channel (HsNaV1.4) (B'SYS GmbH, cat. no. BSYS-NaV1.4-CHO-C).
Authentication	Inhibition of HsNaV1.4 peak currents by STX using QPatch Compact (Sophion Bioscience).
Mycoplasma contamination	B'SYS periodically tests cells for presence of mycoplasma by means of highly sensitive PCR based assays. All delivered cells are free of mycoplasma.
Commonly misidentified lines<br>(See ICLAC register)	N/A

Animals and other research organisms

Policy information about studies involving animals; ARRIVE guidelines recommended for reporting animal research, and Sex and Gender in Research

Laboratory animals	Xenopus laevis (+9 cm, female unknown age).
Wild animals	No wild animals were used in this study.
Reporting on sex	Xenopus oocytes from female frogs were used in this study.
Field-collected samples	No field collected samples were used in this study.
Ethics oversight	Oocytes were harvested from female Xenopus laevis frogs (National Xenopus Resource, Marine Biological Lab) and housed in the UCSF Laboratory Animal Resource Center (LARC) facilities. The use of these Xenopus oocytes was approved by IACUC (protocol approval # AN193390 - 01B) and experiments were performed in accordance with University of California guidelines and regulations.

Note that full information on the approval of the study protocol must also be provided in the manuscript.

Plants

Seed stocks	N/A
Novel plant genotypes	N/A
Authentication	N/A
